# Targeting Pancreatic Cancer Cell Stemness by Blocking Fibronectin-Binding Integrins on Cancer-Associated Fibroblasts

**DOI:** 10.1158/2767-9764.CRC-24-0491

**Published:** 2025-01-31

**Authors:** Chengsheng Wu, Tami Von Schalscha, Diva Sansanwal, Chen Qian, Qinlin Jiang, Ryan M. Shepard, Hiromi I. Wettersten, Stephen J. McCormack, Sara M. Weis, David A. Cheresh

**Affiliations:** 1 Department of Pathology, Moores Cancer Center, Sanford Consortium for Regenerative Medicine at the University of California, San Diego, La Jolla, California.; 2 Alpha Beta Therapeutics, Inc., Claremont, California.

## Abstract

Cancer-associated fibroblasts (CAF) generate an extracellular matrix (ECM) which provides a repository for factors that promote pancreatic cancer progression. In this study, we establish that CAF contribution to pancreatic tumor initiation, i.e., stemness, depends on fibronectin (FN) as a scaffold required for assembly of a collagen-containing fibrotic ECM with a critical dependence on the FN-binding integrins, α5β1 and αvβ3. CAF matrix assembly can be prevented by knockdown of FN, integrin α5, or integrin β3 or by a bispecific antibody with dual recognition of α5β1 and αvβ3 that can also destabilize a preexisting matrix. In mice, the ability of CAFs to produce a stiff collagenous matrix and accelerate tumor initiation can be blocked by knockdown of FN or FN*-*binding integrins or systemic treatment with the α5β1/αvβ3 bispecific antibody. Together, these results reveal that dual targeting of the FN-binding integrins, α5β1 and αvβ3, can block the ability of CAFs and their matrix to enhance pancreatic cancer stemness and progression.

**Significance:**

Simultaneous targeting of two integrins that function as receptors for FN, a protumor ECM protein, can prevent fibroblasts from supporting the malignant behavior of pancreatic cancer cells.

## Introduction

Pancreatic ductal adenocarcinoma (PDAC) has a notoriously dense stroma comprising approximately 80% of the tumor mass that is linked to tumor progression, immune suppression, drug resistance, and metastasis (1–4). This stroma is primarily organized by pancreatic stellate cells that become dysregulated during early tumor development to become cancer-associated fibroblasts (CAF; ref. 5). Intercellular communication between tumor cells and CAFs is a critical enabler of tumorigenesis and progression. In principle, strategies to impair the function of CAFs for cancer therapy include normalizing their function, depletion, or attempting to alter the function of various CAF-produced matrix proteins (6). Yet effectively targeting CAFs *in vivo* has proved to be complicated because of the heterogeneity of CAF phenotypes, populations, and functions (7, 8).

Fibronectin (FN) is a particularly important and impactful component of the stroma that contributes to the lethality of pancreatic cancer (9). Because FN produced by CAFs can assemble on the cell surface to provide a scaffold for the assembly of additional matrix proteins, disrupting FN organization *in vitro* can prevent the formation of a highly fibrotic extracellular matrix (ECM; refs. 10, 11). At the cellular level, integrins clustered on the surface of fibroblasts are receptors that selectively recognize and bind to certain matrix protein ligands, producing a physical link that allows intracellular contractile machinery to impose forces on the matrix proteins (12). Once activated in tumors or fibrotic tissues, fibroblasts gain the expression of two particular FN-binding integrins, α5β1 and αvβ3, that are absent from most normal cell types in the body (12–14). Previous work has established that selective and specific engagement of monomeric FN molecules by integrins αvβ3 and α5β1 is able to transmit mechanical forces that expose cryptic domains required for FN polymerization and the formation of “biologically active fibrils” (15–17). In turn, these fibrils act as a scaffold for the generation of an elaborate matrix that contains a variety of ECM proteins, proteoglycans, and soluble factors that tumor cells can exploit.

During tumor initiation at the primary or metastatic sites, individual tumor cells are forced to overcome a variety of challenging cellular stresses, referred to as isolation stress (18, 19), as they undergo tumorigenesis and attempt to form new proliferative colonies in nonpermissive, inhospitable locales. CAFs provide such tumor cells with nurturing ECM footholds, access to activating secreted factors, and opportunities for intercellular communication. CAFs not only influence tumor cells within the primary tumor but can also gain access to the circulatory system to promote the survival and subsequent growth of circulating tumor cells and facilitate the establishment of new tumor cell outposts by providing a haven for individual tumor cells to seed and survive at metastatic sites (20–22).

Despite recent efforts to characterize the various dysregulated stromal cell types and subtypes, strategies to disempower CAFs have yet to realize success as cancer therapeutics. Our goal was to understand how a pancreatic tumor cell exploits CAFs to facilitate tumor initiation and tumorigenesis to design new strategies to interrupt this cell biological process. Our studies reveal how preventing CAFs from assembling FN fibers can perturb collagen (COL) fibril formation and thereby prevent tumor cells from surviving long enough to “initiate” a new tumor colony. In preclinical PDAC models, genetically or pharmacologically disrupting FN or FN-binding integrins can prevent CAFs from supporting pancreatic cancer cells at different steps of pancreatic cancer progression. Such strategies may have broad application to disempower the activated fibroblasts that exacerbate the progression of other types of cancer and fibrotic disease.

## Materials and Methods

### Reagents, chemicals, and commercial antibodies

Primary antibodies used in this study include FN (E5H6X; Cell Signaling Technology, #26836, RRID: AB_2924220, 1:1,000 for Western blots; 1:200 for IHC), FN [DH1; Novus Biologicals, NBP1-51723, RRID: AB_11059914, 1:200 for immunofluorescence (IF) staining], type I COL α1 chain (COL1A1; E8F4L; Cell Signaling Technology, #72026, RRID: AB_2904565, 1:1,000 for Western blots; 1:200 for IF staining), connective tissue growth factor (CTGF; D8Z8U; Cell Signaling Technology, #86641, RRID: AB_2800085, 1:1,000 for Western blots), vinculin (Boster, #MA1103, RRID: AB_3082541, 1:15,000 for Western blots), GAPDH (D16H11; Cell Signaling Technology, #5174, RRID: AB_10622025, 1:3,000 for Western blots), anti-integrin α5 antibody (P1D6; EMD Millipore, MAB1956Z, RRID: AB_94455, 10 μg/mL for flow cytometry), and CD31 (R&D Systems, #AF3628, RRID: AB_2161028, 1:40 for IHC). Anti-integrin αvβ3 antibody (LM609; 10 μg/mL for flow cytometry) was produced in the Cheresh Lab and is also commercially available (Millipore, MAB1976, RRID: AB_2296419). Anti-integrin α5 was purchased from Millipore (MAB1956). Predesigned siRNAs used in this study were purchased from MilliporeSigma. Each siRNA combo is mixed with two distinct siRNAs (siRNA1 and siRNA2, 1:1 mixture) targeting different gene regions of the gene of interest. The siRNA IDs are listed in Supplementary Table S1.

### Novel antibodies

Novel integrin antibodies provided by Alpha Beta Therapeutics and benchmark controls are summarized in Supplementary Table S2. ABT-101 and ABT-701 are hIgG4-S228P mAbs that recognize human integrins αvβ3 and α5β1, respectively. ABT-601 is a bispecific antibody (BsAb) designed for dual recognition of FN-binding integrins, αvβ3 and α5β1 (ABT-601 is referred to as “BsAb” in this work). ABT-601 comprises the antigen-recognizing Fab domains of ABT-101 and ABT-701 with the same hIgG4-S228P Fc domain.

### Cells

PANC1 human pancreatic cancer cells were obtained from the ATCC (CRL-1469, RRID: CVCL_0480) and cultured using DMEM. Cell line authentication by short tandem repeat analysis was performed for the PANC1 cells in April 2015 and November 2024. KP4 human pancreatic cancer cells were obtained, along with short tandem repeat cell line authentication in January 2023, from the RIKEN BioResource Research Center Cell Bank (RRID: CVCL_1338) and cultured using RPMI. Dr. Andrew Lowy (University of California, San Diego) provided low-passage stock vials of immortalized CAF cell lines hPCF1299 (CAF-1299) and hPCF1424 (CAF-1424) in June 2022 that were previously derived (23) from fresh surgical specimens of human PDAC tissue. All cells were expanded upon receipt, tested for *Mycoplasma* using PCR to detect the 16S rRNA gene from the *Mycoplasma mycoides* cluster (forward 5′-CGA AAG CGG CTT ACT GGC TTG TT-3′ and reverse 5′-TTG AGA TTA GCT CCC CTTCAC AG-3′), and cryopreserved as low-passage stocks (24). For each experiment, stock vials were thawed, cultured for no more than 30 passages, and tested biweekly for *Mycoplasma*.

### Cell-free ECM generation and cell-based assays

Cells were seeded in eight-well chamber slides, glass coverslips, or tissue-treated cell culture plates for up to 7 days to allow cells to produce and deposit sufficient ECM. Decellularization was performed as previously reported (25). Samples were washed twice with Hank’s Balanced Salt Solution (HBSS), incubated for 15 to 20 minutes at 37°C in lysis buffer (8 mmol/L Na_2_HPO_4_ and 1% NP-40, pH 9.6), rinsed three times with wash buffer (10 mmol/L Na_2_HPO_4_ and 300 mmol/L KCl, pH 7.5) and three times with sterile deionized water, and then stored in HBSS. Cell-free ECM was then used for IF staining or other cell-based assays. For cell-based assays, pancreatic cancer cells were seeded atop the cell-free ECM in the presence of 10% or 2% FBS-containing media and grown for 24 hours before cell harvest for Western blot analysis.

### qRT-PCR

RNA was isolated using the RNeasy RNA purification kit (QIAGEN, 75144) following the manufacturer’s instructions. cDNA was synthesized using the High-Capacity cDNA Reverse Transcription Kit (Thermo Fisher Scientific, 4368814), and RT-PCR was performed using CFX96 (Bio-Rad) with SYBR Green (Bio-Rad, 1725272). The custom primer sequences are listed in Supplementary Table S3.

### Mouse study approval

All experiments involving mice were conducted under protocol S05018, approved by the Institutional Animal Care and Use Committee of the University of California, San Diego. All experiments were performed in accordance with the NIH Guide for the Care and Use of Laboratory Animals. All animals were housed under standard conditions, i.e., given unrestricted access to food and water, housed in standard cages, and rooms regulated to control temperature and light cycles. Cell lines used for *in vivo* experiments confirmed negative for a panel of human pathogens.

### Subcutaneous xenograft model

The number of PANC1 cells injected varies greatly among studies (26–28), and this is likely influenced by the use of various strains of immune-compromised mice. Because nu/nu mice have a relatively less compromised immune system than NOD-SCID or NOD/SCID gamma strains, we selected an injection amount of 0.5 million cells for the experiment in Fig. 5A. Human PANC1 cells of 5 × 10^5^ were mixed with or without an equal number of human CAF-1299 cells transfected with different siRNAs for 72 hours. Knockdown was verified via Western blot. For the antibody treatment groups, PANC1 or CAF-1299 cells were premixed with 10 μg/mL antibody for 10 minutes before injection. Cells were suspended in a 1:1 mixture of HBSS and phenol red–free basement membrane matrix (BD Biosciences; total volume is 100 μL per injection) and injected subcutaneously in 6- to 8-week-old female immune-compromised nu/nu mice (Charles River Laboratories, #088, RRID: IMSR_CRL:088). Fresh antibody (10 mg/kg) was injected intraperitoneally twice a week, and mice were examined twice weekly for palpable tumors. A tumor larger than 100 mm^3^ in volume is counted.

### Orthotopic pancreatic cancer model

**Figure 1:**
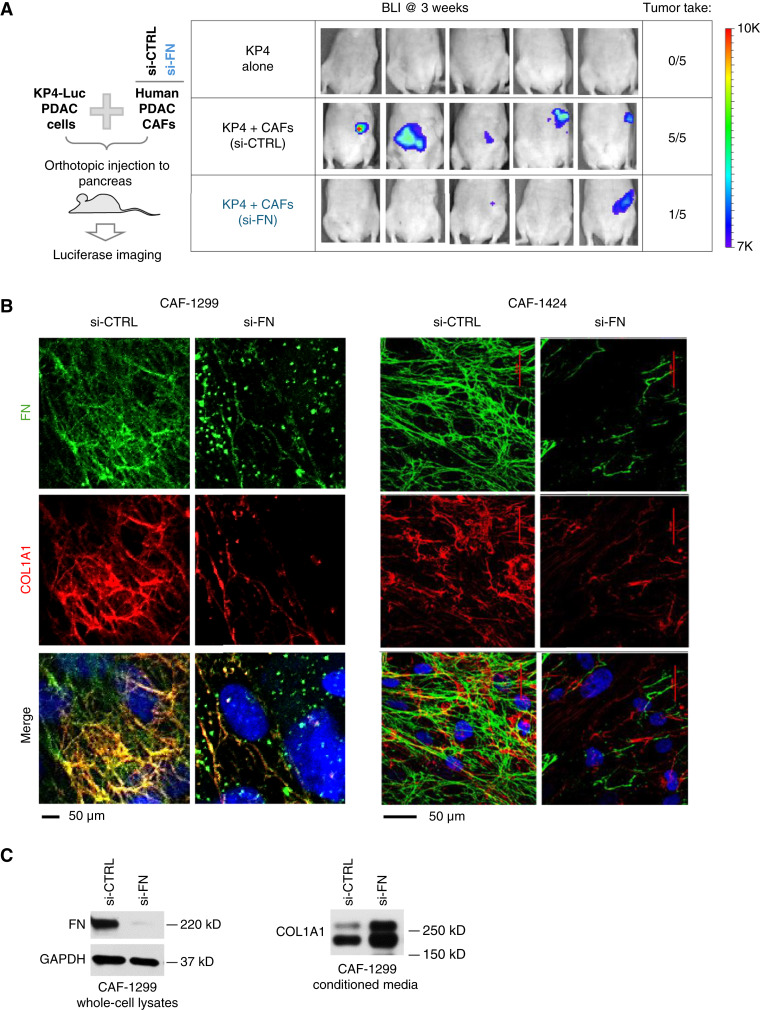
Tumor cells utilize CAF-produced FN to overcome isolation stress. **A,** CAFs enhance tumor initiation via FN (orthotopic pancreas cancer model). Luciferase-expressing KP4 PDAC cells were orthotopically injected (with or without CAF-1299) into the pancreas of nu/nu mice. After 3 weeks, luciferase imaging was performed to survey tumor establishment in the pancreas. **B,** FN knockdown prevents CAF assembly of COL fibers. CAF-1299 and CAF-1424 cells were treated with siRNA for a scramble control vs. FN. After 72 hours, IF staining shows FN and COL content. Images are representative of at least three independent experiments. **C,** Knockdown of FN in CAFs increases soluble COL in media. CAF-1299 cells were cultured for 72 hours, and then the conditioned media and cell lysates were collected and processed for immunoblotting to confirm FN knockdown and assess the level of soluble COL secreted by the cells into the media. si-CTRL, siRNA Universal Negative Control; si-FN, siRNA-mediated knockdown of *FN1*.

To determine an injection number that would allow us to assess changes in tumor initiation at a relatively early timepoint, we performed a pilot study to compare orthotopic injections of 5, 1, or 0.2 million KP4-Luc cells. Although no tumors were detected by bioluminescence imaging (BLI) after 5 weeks, imaging at 10 weeks revealed tumors in three of three mice that received five or one million KP4-Luc cells but only one of three mice for the 0.2 million cell group (Supplementary Fig. S1A). We reasoned that orthotopic implantation of one million KP4-Luc cells should produce tumors in all mice that are detectable after 5 to 10 weeks, and furthermore, that co-injection with CAFs should accelerate tumor initiation (i.e., detection at timepoints less than 5 weeks). For the experiment shown in Fig. 1A, 1 × 10^6^ human KP4 cells stably transfected with luciferase lentivirus (KP4-Luc) were mixed with or without the equal number of human CAF-1299 cells transfected with different siRNAs for 72 hours. Knockdown was verified via Western blot analysis. Cells were suspended in HBSS and injected into the pancreas of 6- to 8-week-old female immune-compromised nu/nu mice (Charles River Laboratories, #088, RRID: IMSR_CRL:088). The tumor growth was monitored twice a week using noninvasive BLI and an IVIS Spectrum system (PerkinElmer). All mice were imaged 10 minutes after being injected with D-luciferin (L9504, Sigma-Aldrich). Immunoblotting to assess the duration of siRNA-mediated knockdown of FN1 for CAFs (Supplementary Fig. S1B) shows that FN knockdown remained effective after 6 days in culture but significantly declined by day 14.

### IF staining and confocal microscopy

CAFs were seeded on an eight-well chamber slide (Nunc Lab-Tek chamber slide; Thermo Fisher Scientific) overnight. The next day, cells were treated with siRNA targeting the genes of interest or antibodies (10 μg/mL) for 72 hours and then fixed with 4% paraformaldehyde for 15 minutes at room temperature. To target the preexisting matrix, CAFs were seeded and allowed to deposit the matrix for 72 hours. At 72 hours, the antibody was added (10 μg/mL) and allowed to incubate for an additional 72 hours. The cells and matrix were then fixed with 4% paraformaldehyde for 15 minutes at room temperature. All instances were followed by 45 minutes of incubation with a blocking buffer containing HBSS or HBSS/Tween 20 (0.2%) supplemented with 5% horse serum. The cells were then incubated with primary antibodies at a dilution of 1:200 (anti-FN or COL1A1) overnight at 4°C, followed by secondary antibodies at a dilution of 1:1,000 for 1 hour and 4',6-diamidino-2-phenylindole (DAPI) staining for 5 minutes at room temperature. Images were acquired by a Nikon Eclipse Ti C2 confocal microscope with multiple Z-stack images and analyzed with NIS-Elements Viewer 5.21. The fluorescent signal was quantified as % area fraction using ImageJ.

### Immunoblotting

Immunoblotting was performed as previously described (29). Briefly, cells were washed twice with HBSS before lysing with 1× RIPA buffer containing protease and phosphatase inhibitors, 2× sample buffer containing 1× reducing agent, or, for the conditioned supernatant, 4× sample buffer containing 1× reducing agent (Bio-Rad, #1610737 and #1610747). A bicinchoninic acid (BCA) protein assay (Thermo Fisher Scientific, 23227) was performed, and the lysates were normalized. Sample buffer (NuPAGE LDS Sample Buffer 4×, Sigma, #NP0007) and reducing agent (NuPAGE Sample Reducing Agent, Sigma, #NP0009) were added to the cell lysates. All samples were heated at 95°C for 5 minutes. Ten micrograms of protein or 30 μL of each sample containing Laemmli buffer was loaded onto an SDS-PAGE gel. Blocking was performed using 5% BSA in Tris-buffered saline with Tween-20 (TBS-T), and probing was performed using 5% BSA in TBS-T buffer.

### Flow cytometry

Cell pellets were washed with PBS, blocked with 1% BSA in PBS for 30 minutes at room temperature, and stained with or without indicated primary antibodies with fluorescently labeled secondary antibodies. Cells were incubated with a LIVE/DEAD Fixable Blue Dead Cell Stain Kit (Invitrogen, L23105). Flow cytometry was performed on a BD Fortessa X-20 (BD Biosciences) analyzer, and the data were analyzed using FlowJo (TreeStar) software.

### IHC

IHC staining was performed on formalin-fixed, paraffin-embedded slides using an ImmPRESS Excel Staining Kit (VectorLabs, MP-7602) following the manufacturer’s instructions. For integrin human FN (hFN), low-pH antigen retrieval (Invitrogen, 00-4955-58) was performed for 40 minutes at 95°C. For CD31, high-pH antigen retrieval (Invitrogen, 00-4956-58) was performed for 40 minutes at 95°C. Mason's trichrome staining was performed using Abcam AB150686. Picrosirius red staining was performed using Abcam AB246832. The slides were imaged using an Olympus VS200 slide scanner (Olympus) at the University of California San Diego School of Medicine Microscopy Facility (funded by core grant NINDS P30NS047101). Scanned images were analyzed for protein expression as the area fraction per area of tumor tissue calculated using QuPath, open-source software for bioimage analysis (30).

### Atomic force microscopy

Atomic force microscopy measurements were performed using an MFP-3D atomic force microscope (Asylum Research). Silicon nitride cantilevers were used with a normal spring constant of 0.08 Nm^−1^ and a 200 μm length (NanoWorld, PNP-TR-50). Cantilevers were calibrated using the thermal fluctuation method and verified by probing glass of known elasticity. The specimens used were 20-μm-thick frozen sections of human PDAC tissue embedded in Optimal cutting temperature (OCT) compound. Each tissue section was thawed and equilibrated to room temperature by immersion in HBSS for 5 minutes. Indentation tests for the specimens were carried out at a 2 μm per second loading rate to generate 16 force curves across equally distributed regions of 20 × 20 μm. Young’s moduli of the samples were determined by fitting force curves with the Hertz model using a Poisson ratio of 0.5.

### Data availability

The data generated in this study are available upon request from the corresponding author.

## Results

### Tumor cells benefit from CAF-produced FN to overcome isolation stress

FN plays a critical role in ECM assembly and remodeling during a diverse range of physiologic and pathologic responses. To gauge the importance of CAF-produced FN during tumor initiation, KP4 human PDAC cells were injected orthotopically into the pancreas of immune-compromised nu/nu mice alone or at a 1:1 ratio with PDAC-derived CAFs to evaluate tumor initiation in the pancreas (Fig. 1A). After 3 weeks, BLI revealed *bona fide* tumors (luciferase signal >1e+06) in five of five mice, compared with zero of five mice injected with KP4 cells alone, validating the CAF dependence of tumor initiation for this model. In contrast, CAFs with siRNA-mediated knockdown of FN could enable tumor initiation, a hallmark of tumor stemness, in only one of five mice, suggesting that CAF-produced FN could largely account for the contribution of CAFs during this process. Because the siRNA-mediated knockdown efficiency begins to decline 6 days after transfection (Supplementary Fig. S1B), these data suggest that CAF-produced FN is critical to enable the early steps of tumor initiation *in vivo*.

### CAF interaction with FN provides a critical scaffold for the deposition of COL fibers

Previous work has identified FN as a scaffold that coordinates the assembly and incorporation of additional proteins and secreted factors into the ECM (11). Indeed, CAFs isolated from patients with PDAC generate a fibrotic ECM in which FN and COL fibers clearly show a high degree of co-patterning (Fig. 1B; Supplementary Fig. S2A). As evidence for FN’s role as a critical scaffold for the construction of additional matrix proteins, siRNA-mediated knockdown of FN1 prevents CAFs from generating COL fibers (Fig. 1B). This reduced assembly of COL into insoluble fibers is consistent with the increased content of “soluble COL” in conditioned media collected from CAFs with FN knockdown compared with scramble control (Fig. 1C). In contrast, knockdown of *COL1A1* does not affect FN fiber assembly (Supplementary Fig. S2B), highlighting the unique role of FN as a critical base scaffold upon which additional ECM components such as COL are layered. Together, these findings demonstrate not only that FN is required for the CAF-mediated assembly of COL into fibers but also that the genetic approach to disrupting this process establishes proof of principle for target tractability.

### FN-binding integrins are critical for fibrotic matrix assembly

**Figure 2:**
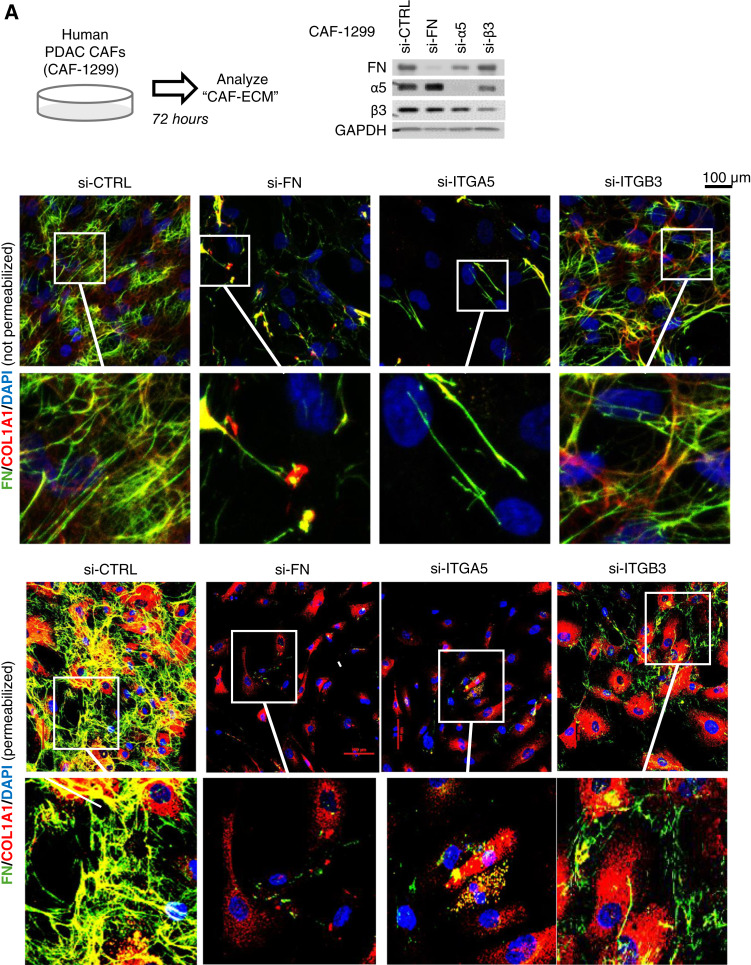
FN-binding integrins mediate ECM production. Knockdown of FN or FN-binding integrins in CAFs prevents the formation of FN and COL fibers. CAF-1299 cells were treated with siRNA for a scramble control vs. FN, *ITGA5*, or *ITGB3*. After 72 hours, IF staining shows FN and COL content. The top set of images were not permeabilized. Images are representative of at least three independent experiments. Blots confirm knockdown. si-CTRL, CAFs with siRNA for a Universal Negative Control; si-FN, CAFs with siRNA-mediated knockdown of *FN1*; si-α5, CAFs with *ITGA5* knockdown; si-β3, CAFs with *ITGB3* knockdown.

Previous studies have established that activated fibroblasts (myofibroblasts) use cell surface integrins as anchors to apply tension to FN molecules, revealing cryptic sites required for polymerization into FN “fibers” that, in turn, provide a physical scaffold and repository for the assembly and recruitment of additional procancer factors to support the invasive behavior of cancer cells (10, 15–17). Although several integrins can serve as receptors for FN, integrins αvβ3 and α5β1 are absent on normal cells but become upregulated on a variety of activated cell types, including myofibroblasts (31–33). Indeed, CAFs isolated from human PDAC tumors show robust protein expression of both the α5 and β3 subunits (Fig. 2). Similar to knockdown of *FN1*, COL fibrillogenesis can be reduced by knockdown of the integrin β3 (*ITGB3*) subunit (in which expression is the limiting factor for the formation of the αvβ3 heterodimer) or the integrin α5 (*ITGA5*) subunit (in which expression similarly dictates α5β1; Fig. 2). Imaging permeabilized cells reveals that knockdown of FN or FN-binding integrins does not eliminate the ability of CAFs to produce COL (as evidenced by the presence of intracellular COL), but rather that the ability of CAFs to *assemble* extracellular COL into fibers has been largely eliminated (Fig. 2). Together, these findings support the notion that FN–integrin interactions on the surface of CAFs are required for the formation of the FN fibers that function as a scaffold for other fibrotic matrix proteins and furthermore suggest that targeting integrins α5β1/αvβ3 may provide an opportunity to significantly impair the contribution of CAFs to cancer progression. Because αvβ3 and α5β1 integrins are generally absent on normal cell types but highly expressed on CAFs, targeting their function may provide an opportunity to selectively suppress the pathologic fibrosis that supports PDAC progression.

### Integrin-blocking antibodies prevent and reverse CAF assembly of ECM

**Figure 3:**
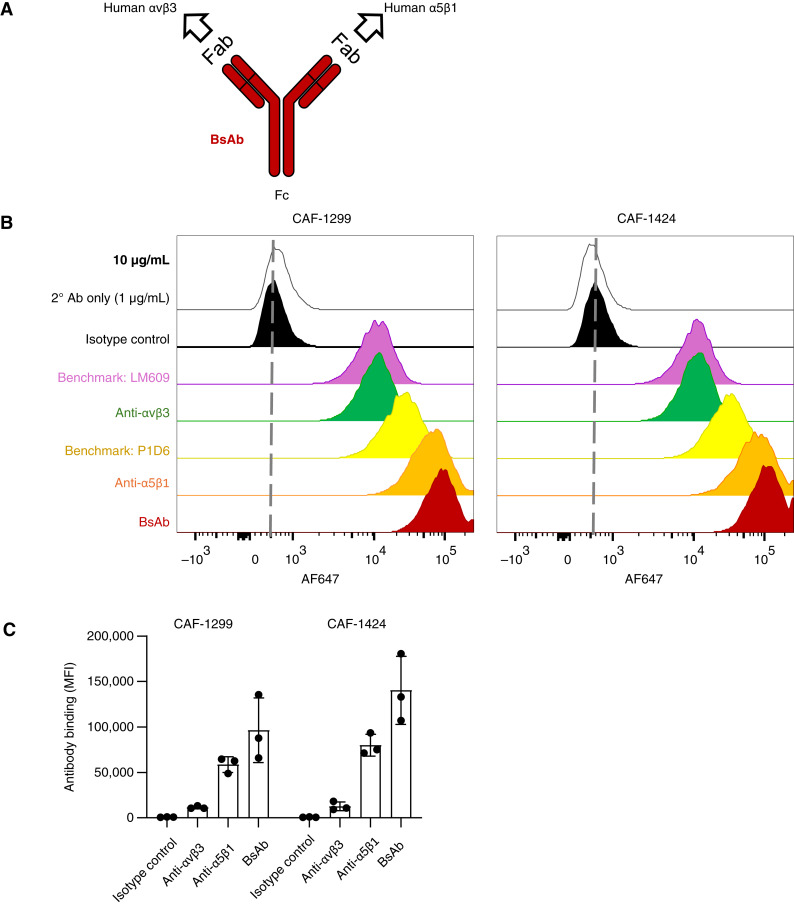
BsAb improves the targeting of FN-binding integrins on CAFs. **A,** BsAb for dual recognition of αvβ3 and α5β1 heterodimers. Schematic depicts the design of a novel BsAb with monovalent recognition of two antigens, integrins αvβ3 and α5β1. The Fab domains for the BsAb are identical to the “control” bivalent (i.e., monospecific) mAb recognizing αvβ3 (derived from etaracizumab) and α5β1 (derived from volociximab). **B,** BsAb binding to cells compared with commercial and control antibodies. Flow cytometry plots show the binding of each antibody to CAF-1299 and CAF-1424. **C,** BsAb shows improved binding to CAFs compared with constituent mAbs. The graph shows median fluorescence intensity (MFI) for antibody binding to CAF-1299 and CAF-1424 cells. Bars and error bars represent SD from *n* = 3 independent experiments.

To simultaneously target both integrins required for ECM production by CAFs using a single agent, we designed a novel BsAb for dual monovalent recognition of the integrin α5β1 and αvβ3 heterodimers (Fig. 3A). We first compared this BsAb with its two parental control bivalent mAbs that individually recognize integrins αvβ3 or α5β1. Commercially available antibodies recognizing integrin αvβ3 (LM609) and *ITGA5* (P1D6) were included as additional benchmarks. Flow cytometry analysis was performed to evaluate the binding of each antibody (tested at a concentration of 10 μg/mL) to two different patient-derived CAF lines of PDAC, CAF-1299 and CAF-1424. For both CAF models, the median fluorescence intensity signal for the BsAb was slightly more than the additive sum of its two parental mAbs (Fig. 3B and C).

**Figure 4:**
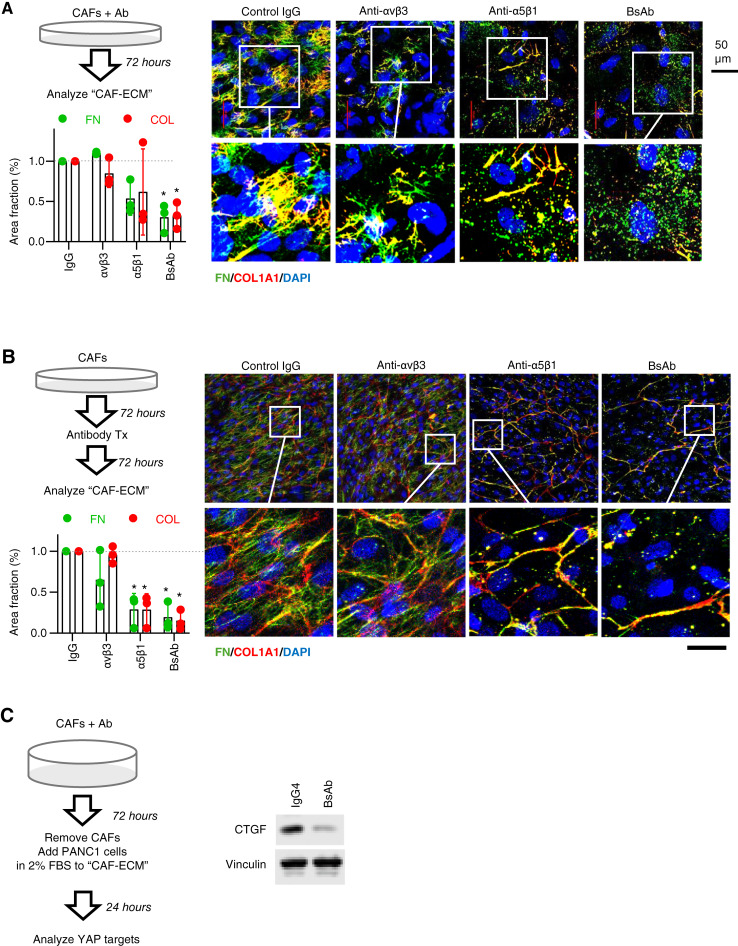
Dual blockade of integrins αvβ3/α5β1 can prevent and reverse CAF-ECM assembly. **A,** Function-blocking antibodies targeting FN receptors prevent CAF assembly of FN/COL fibers. CAF-1299 cells were incubated with control IgG vs. indicated antibodies for 72 hours and then processed for immunostaining to examine FN and COL. Images are representative of at least three independent experiments. The graph shows the quantification of staining as mean ± SD for each marker that was measured as % area for each experiment and then normalized to IgG control. *, *P* < 0.05 using one-sample *t* test. **B,** Integrin-targeted antibody can disrupt preexisting CAF-produced ECM. CAF-1299 were plated and allowed to produce ECM for 72 hours before adding control IgG vs. indicated antibodies for an additional 72 hours. Samples were then processed for immunostaining to examine FN and COL. The graph shows the mean ± SD staining for each marker measured as % area in each experiment and normalized to IgG control. *, *P* < 0.05 using the one-sample *t* test. **C,** BsAb prevents upregulation of connective tissue growth factor (CTGF), a protein that regulates cell proliferation, migration, and adhesion. CAF-1299 cells were plated and allowed to produce ECM for 72 hours and then treated with antibody for another 72 hours before cells were removed to leave behind a cell-free ECM atop which PANC1 cells were then plated. After 24 hours, the PANC1 cells were lysed and prepared for immunoblotting to detect the protein expression of CTGF. Blots are representative of at least three independent experiments. Tx, treatment.

Consistent with the effects of genetic knockdown of either β3 or α5, treating CAFs with the mAbs recognizing αvβ3 or α5β1 shows a variable ability to reduce the assembly of FN and COL fibers that may reflect the relative expression level of these integrins in a given cell population, whereas the BsAb with dual recognition of integrins αvβ3/α5β1 produces a more complete blockade of FN and COL fibril formation than either mAb alone (Fig. 4A). Immunostaining CAFs for FN and actin reveals punctate FN expression in the antibody-treated cells that is no longer aligned with actin fibers, confirming that this antibody does not affect the matrix adhesion and cytoskeleton of these cells (Supplementary Fig. S3A). Decellularization (i.e., removal of CAFs) after 72 hours of treatment leaves behind a “CAF-ECM” substrate that clearly illustrates the significant effect of the BsAb on both FN and COL fibers (Supplementary Fig. S3B). A similar effect of the BsAb is observed for primary hepatic stellate cells isolated from patients with liver fibrosis or primary lung fibroblasts isolated from patients with fibrotic lung disease (Supplementary Fig. S4), suggesting that this antibody may have the potential for broad use as a general antifibrotic agent to target activated fibroblasts that depend on FN and integrins α5β1/αvβ3 for matrix assembly. Consistent with a change in matrix assembly but not production, treating CAFs with the BsAb does not alter mRNA expression of either *FN1* or *COL1A1* genes (Supplementary Fig. S5).

To directly compare the BsAb with a combination of monospecific mAbs *in vitro*, FN immunostaining was evaluated for CAFs subjected to antibody treatment for 72 hours. Under these *in vitro* conditions for which cells are exposed to saturating antibody doses, all of the test antibodies equivalently block CAF production of FN compared with the IgG isotype control (Supplementary Fig. S6). Interestingly, although the αvβ3 mAb produces a significant reduction in FN levels, it does not induce the unique FN puncta as observed for the α5β1 mAb, BsAb, and combination of α5β1/αvβ3 mAbs.

Integrins exist in a dynamic equilibrium of conformational states such that binding events are continuously being formed and dissociated. Although small peptide Arg-Gly-Asp–based competitive inhibitors cannot easily reverse integrin–ligand binding, a function-blocking β1 antibody was reported to increase the dissociation rate of integrin–FN complexes and act allosterically (34). To test if the BsAb may be able to interfere with a preexisting matrix, we allowed CAFs to deposit ECM proteins for 72 hours in culture before adding integrin-targeted antibodies. Indeed, over the span of 3 days, the BsAb was able to disrupt preexisting FN and COL fibers produced by CAFs (Fig. 4B), suggesting that its ability to allosterically interfere with continuously cycling on/off states of FN–integrin binding can exert a significant antifibrotic effect by shifting the equilibrium to a nonadhesive state.

As one biological readout for how PDAC cells respond to CAFs, we considered the ability of CAF-produced ECM to upregulate tumor cell expression of CTGF, a YAP target gene and matricellular protein produced by both stromal and tumor cells that mediates their cross-talk, promotes fibrosis, and enhances tumor initiation in pancreatic cancer (35, 36). CAFs were cultured for 72 hours to allow for matrix deposition and then were treated for an additional 72 hours with isotype control or the BsAb. CAFs were removed to leave behind “CAF-ECM” upon which PANC1 cells were plated. After an additional 24 hours, PANC1 cell lysates were collected and analyzed by immunoblotting. In this model, CAF-ECM triggers a strong upregulation of CTGF, a protein that regulates cell proliferation, migration, and adhesion, and this is prevented in the presence of the BsAb (Fig. 4C). This assay demonstrates how blocking the two primary FN-binding integrins, αvβ3 and α5β1, can prevent tumor cell upregulation of CTGF in response to CAF-produced ECM.

### Disrupting FN-binding integrins prevents the ability of CAFs to enhance tumor initiation

**Figure 5:**
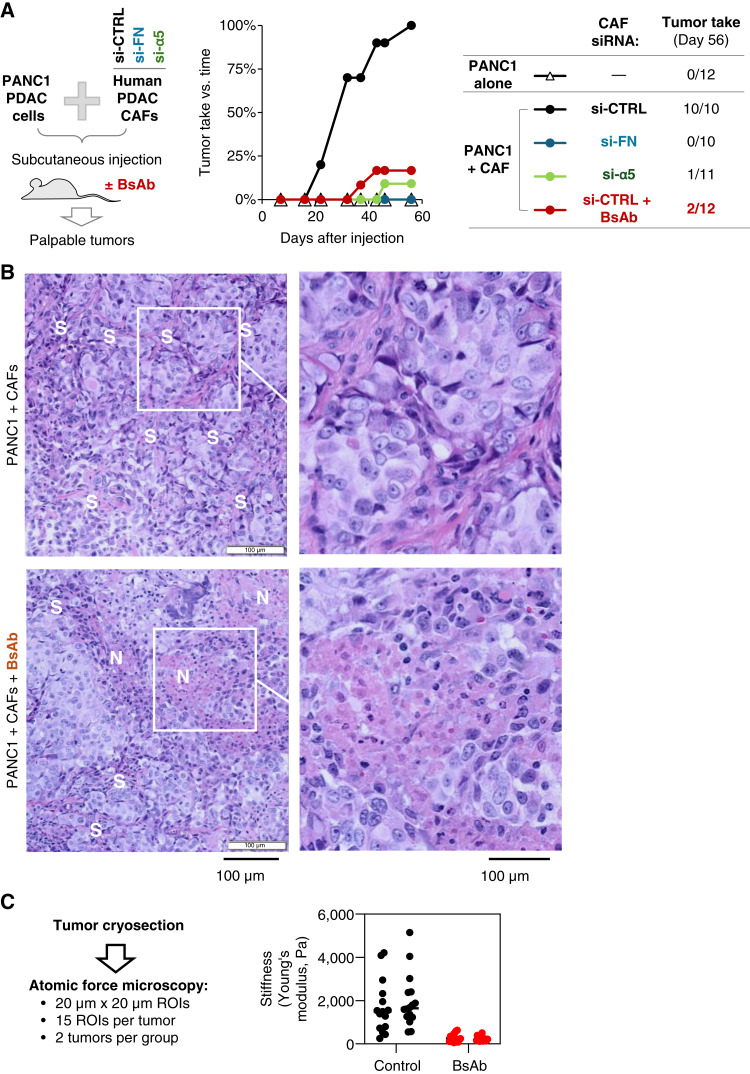
Disrupting FN-binding integrins prevents the ability of CAFs to enhance tumor initiation. **A,** CAFs enhance tumor initiation via FN and FN-binding integrins (subcutaneous xenograft model). PANC1 human PDAC cells were injected subcutaneously (with or without CAF-1299) into the flank areas of nu/nu mice. Mice were monitored twice weekly to detect the earliest emergence of palpable tumors. The graph shows tumor take rate vs. time for 10–12 mice per group, using a volume of 100 mm^3^ (computed as length × width2) as the threshold for tumor take. At the endpoint of the experiment (day 56), tumors were harvested and prepared for histologic analysis. **B,** BsAb treatment reduces tumor stroma and increases necrosis. Tumor sections were stained using hematoxylin and eosin. Areas of stroma (S) and necrosis (N) are noted. **C,** BsAb treatment reduces tumor stiffness. Cryosections of tumors were analyzed using atomic force microscopy to evaluate tissue stiffness. Dots depict the mean value for each 20 × 20 μm region of interest (ROI). Fifteen ROIs were evaluated per tumor, for two tumors per group.

To evaluate the ability of the αvβ3/α5β1 BsAb to disrupt tumor initiation *in vivo*, we utilized a subcutaneous xenograft model to readily detect the earliest emergence of tumor initiation over time. As for the orthotopic model (Fig. 1A), a suboptimal number of PDAC cells were injected so that initiating a tumor depended on the co-injection of CAFs. In this model, no palpable tumors were detected 8 weeks after subcutaneous injection of a limiting number of PANC1 human PDAC cells alone, whereas co-injection of PANC1 cells with CAFs at a 1:1 ratio produced a 100% take rate (Fig. 5A). That is, palpable tumors formed at 12 of 12 injection sites co-injected with PANC1 cells with CAFs, validating this model as a readout for CAF-dependent tumor initiation.

As observed for the orthotopic model (Fig. 1A), the “boost” in CAF-dependent tumor initiation at a subcutaneous site could be prevented using CAFs with siRNA-mediated knockdown of *FN1*, whereas CAFs with *ITGA5* knockdown produced tumors at 1 of 11 injection sites. Because the siRNA knockdowns are transient, these results suggest that the CAF contribution to tumor initiation occurs within the first several days after co-injection when tumor cells exploit CAFs to overcome isolation stress as they colonize a tumor-initiating niche. During this critical phase, eliminating CAF expression of FN or *ITGA5* is sufficient to completely account for their ability to boost tumor initiation.

Accordingly, we asked if the therapeutic BsAb designed for dual targeting of integrins αvβ3/α5β1 could exert a similar activity to block tumor initiation. Because this BsAb recognizes antigens on most species except mice, its influence on tumor initiation in this xenograft model can be attributed to its direct binding to its antigens on the human CAFs co-injected with human PDAC cells but not to the integrins on the surface of mouse stromal or vascular cells. Tumor cells and CAFs were premixed with 10 μg/mL BsAb immediately before injection. Once the tumor cells were injected, the antibody was then administered systemically by intraperitoneal injection twice weekly for the experiment at a dose of 10 mg/kg. Remarkably, mice treated with the αvβ3/α5β1 BsAb developed palpable tumors at only 2 of 12 injection sites (Fig. 5A), suggesting that the boost in tumor initiation offered by CAFs can be targeted therapeutically. Because the effects of the BsAb are mimicked by the knockdown of FN or α5 in the CAFs, we propose that blocking integrin function on CAFs has the ability to prevent tumor initiation.

### BsAb treatment reduces the fibrotic effect of human CAFs co-injected with tumor cells

To evaluate the mechanism(s) of action for the knockdown and blockade strategies, all palpable tumors were harvested at the 8-week endpoint of the experiment. Compared with untreated tumors, analysis of hematoxylin and eosin–stained sections show far less stroma but display extensive necrosis in the two tumors that formed in the BsAb-treated group (Fig. 5B). Previous studies have documented that PDAC tumors are highly stiff, primarily because of the extensive stroma and ECM deposited within the tumor, and that stiffness correlates with PDAC progression in mice and man (37, 38). Consistent with this, mice treated with the BsAb show significantly lower tissue stiffness throughout the entire tumor as measured by atomic force microscopy (Fig. 5C).

**Figure 6:**
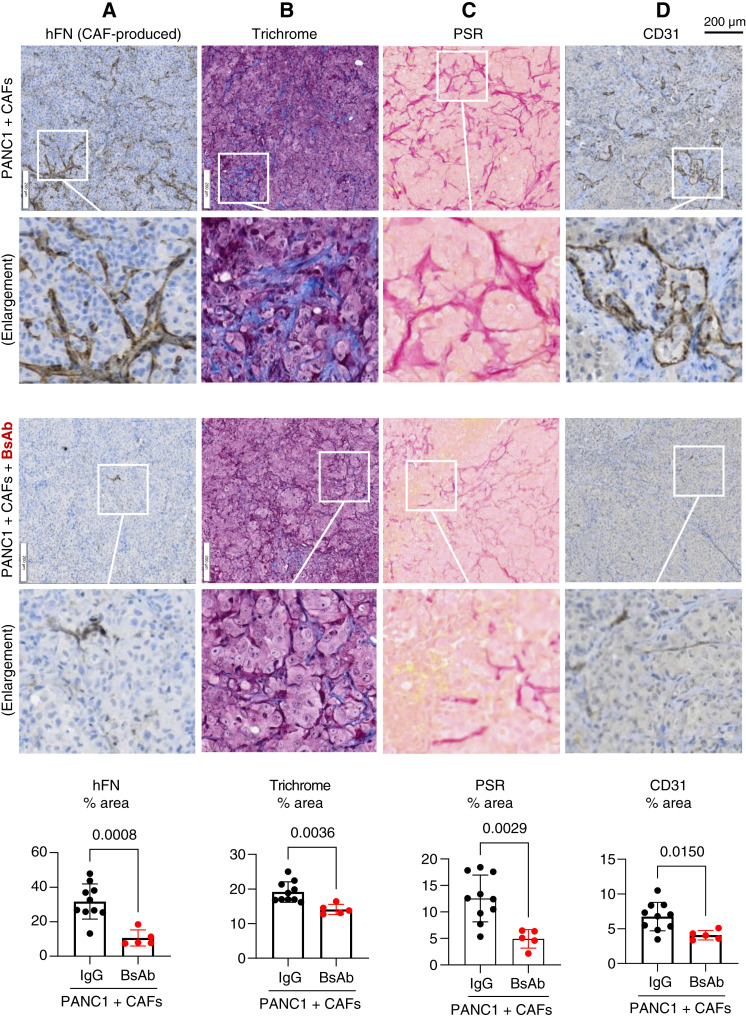
BsAb treatment reduces the fibrotic effect of human CAFs co-injected with tumor cells. **A–D,** BsAb-treated tumors contain less CAF-produced FN, fibrosis, and angiogenesis. Serial formalin-fixed, paraffin-embedded sections of tumors were processed for IHC detection of hFN (**A**) or CD31 (**D**), shown in brown. Mason's trichrome (**B**) and picrosirius red (PSR; **C**) histologic stains were used to visualize COL. Graphs depict the quantification of IHC staining using QuPath, with each dot representing the mean value for each tumor slice examined. *P* values were computed using the Student *t* test.

Analysis of the tumor microenvironment also supports the notion that the co-injection of CAFs produces a highly fibrotic and reactive tumor environment that can be targeted therapeutically. Staining tumors formed by co-injection of PANC1 + CAFs using an antibody that recognizes hFN, but not mouse FN, reveals areas of intense fibrillar hFN staining (Fig. 6A), suggesting that the co-injected CAFs represent a significant producer of tumor stroma in this model relative to the host (i.e., mouse) fibroblasts. Tumor areas with dense hFN staining also show significant fibrosis (COL polymerization), as evaluated by Masson’s trichrome (Fig. 6B) and picrosirius red staining for COL fibers (Fig. 6C). In comparison, tumors from mice treated with the BsAb show a complete absence of hFN, whereas COL fibers seem both fewer and smaller. Because the BsAb utilized in this study does not recognize mouse antigens, it can identify only the injected human PDAC cells and human CAFs in this xenograft model. This particularity suggests that interactions between co-injected PANC1 cells and CAFs mediate the earliest steps of tumor initiation before any mouse stromal cells are recruited into the tumor microenvironment. Analysis of serial sections of tumors stained for hFN and COL suggests that the human CAFs contribute to the production of a highly fibrotic matrix, whereas mouse host cells that are insensitive to the BsAb may be the source of less developed COL networks.

In addition to the observed suppression of CAF-generated FN, we predict that in humans, the BsAb might gain additional antitumor efficacy by acting on activated cell types within the tumor that are known to gain expression of αvβ3/α5β1, including angiogenic endothelial cells and tumor-associated macrophages. In the xenograft model, we observed a reduction in immunostaining for CD31, an endothelial cell marker used to assess tumor angiogenesis. It is therefore possible that the BsAb may exert an indirect antiangiogenic effect on the mouse vascular compartment (Fig. 6D), by inhibiting the accumulation of stimulatory factors and ECM proteins within the tumor microenvironment that stimulate vascular cells to generate new blood vessels (39, 40).

## Discussion

CAFs are dysregulated stromal cells that exert a massive influence on the biology of tumor cells to facilitate the progression of many solid tumor types, especially PDAC with its notorious dense and reactive stroma. Despite efforts to characterize various CAF types and subtypes (41), strategies to “disempower” CAFs have yet to realize success as cancer therapeutics. In addition to their profibrotic role within the primary tumor microenvironment, CAFs can gain access to the circulatory system to promote the survival and subsequent growth of circulating tumor cells and facilitate the establishment of new tumor cell outposts by serving as the “soil” or “niche” that creates a friendly environment for individual tumor cells to seed and survive at distant sites (20, 42). Pancreatic cancer is notorious for its dense and stiff stroma that supports tumor progression and hinders the delivery of therapeutic drugs (2). Although tumor cells within this oasis benefit from a plethora of growth factors and stimulatory signals that allow their uncontrolled growth and survival, we focus on how CAFs may nurture individual tumor cells during tumor initiation, a situation which may also occur during metastatic colonization of distant sites where tumor stroma has yet to form. We consider how cross-talk between CAFs and tumor cells represents a limiting factor required for tumor cells to survive the challenges encountered during what we refer to as “isolation stress” (18). This relates not only to the ability of limited numbers of cells to establish tumor colonies within the pancreas but also to the situations faced by circulating tumor cells or disseminated tumor cells that need help from extrinsic factors in order to survive in the circulation and to initiate tumor formation at distant sites where they are surrounded by normal tissue and subjected to immune surveillance.

Our work demonstrates that preventing CAFs from generating cell surface FN fibers can have significant effects, akin to destroying the foundation of a building. Similar effects have been reported for agents such as the functional upstream domain “FUD” peptide (and a PEGylated form developed to slow its rapid renal clearance; ref. 43) that binds to the N-terminal domain of FN to sterically inhibit the polymerization of FN monomers into fibrils. Although such agents that block FN fibrillogenesis have been shown to produce significant antifibrotic activity, pharmacokinetic stability issues have limited their development for use in man. In contrast, we have devised an antibody therapeutic to disrupt FN fibrillogenesis by interfering with the anchoring of FN to FN-binding integrins, thereby preventing the tensile forces that reveal cryptic sites required for FN polymerization. We show that dual blockade of αvβ3/α5β1 or integrin knockdown can inhibit CAFs from producing fibrillar FN and COL, and this prevents stem-like reprogramming of tumor cells, measured here as tumor initiation in mice. Because the BsAb selectively targets human (but not mouse) cells, it provides a unique mechanistic tool that could be used in future studies to dissect the benefits of perturbing integrin function on CAFs alone, tumor cells alone, or both cell types.

**Figure 7:**
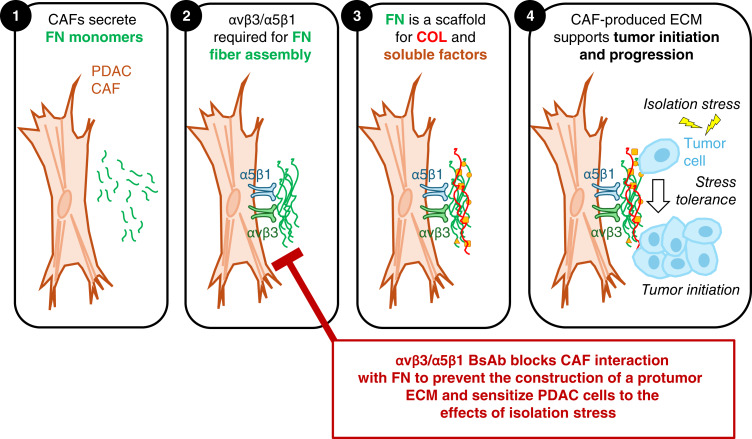
CAF-produced FN boosts tumor initiation (summary schematic). CAFs produce a dense and reactive stroma that supports tumor initiation and progression. This project establishes the link between cell surface integrins and FN as a lynchpin for the construction of protumor ECM that can account for the ability of CAFs to support tumor initiation for multiple *in vitro* and *in vivo* models. Knockdown or antibody blockade of integrins αvβ3 and/or α5β1 can disrupt FN fiber assembly. FN fibers are known to function as a scaffold that mediates the assembly of other ECM proteins and the incorporation of protumor-secreted factors. Knockdown of αvβ3/α5β1 or their dual blockade using a novel BsAb can disrupt this cascade by preventing the initial assembly of FN fibers. This approach has broad potential as a novel strategy to target the aberrant fibrosis that exacerbates the progression of cancer and fibrotic disease.

Our work reveals that CAF-mediated assembly of FN and COL fibers is especially enabling and enhancing when PDAC cells are challenged with “isolation stress,” a state that occurs during various aspects of tumor initiation and progression. We show that disrupting the ability of CAFs to organize FN fibers results in the loss of COL fibril formation and thus provides an opportunity to interfere with intercellular communication and prevent tumor cell survival long enough to “initiate” a new tumor colony (Fig. 7). Though not addressed directly in this study, it is tempting to speculate that tumor initiation at a metastatic site would similarly depend on the CAF-produced matrix. We show that disrupting tumor initiation can be achieved by knocking down the expression of FN or its receptors or by utilizing a unique BsAb that targets the two primary receptors CAFs use to organize FN, integrins αvβ3 and α5β1. By perturbing the ability of CAFs to generate the FN fibrils that function as a cornerstone for the construction of additional ECM components, tumor cells are less able to overcome the effects of isolation stress. As such, disrupting FN–integrin binding provides a powerful approach to prevent CAFs from supporting PDAC tumor initiation.

The therapeutic efficacy of blocking FN-binding integrins may be derived from direct effects on CAFs plus indirect effects on various cell types that are sensitive to changes in the tumor stroma. Aside from CAFs, αvβ3 and α5β1 are upregulated on several key cell types in a tumor, including mesenchymal tumor cells and angiogenic endothelial cells. For example, the CAF matrix is a rich source of VEGF and other factors that promote tumor angiogenesis (39, 40). By targeting αvβ3/α5β1 on tumor-associated CAFs, we observed that the level of CD31-positive vascular cells was significantly reduced, representing an example of an indirect effect of the human-specific BsAb that cannot directly recognize mouse vascular cells. By preventing the deposition of a dense, reactive ECM that contributes to an immunosuppressive environment, targeting CAFs with an agent like the αvβ3/α5β1 BsAb may also improve tumor responsiveness to immunotherapy. Reducing the extent of fibrotic tumor stroma may also improve blood flow and the delivery of systemically administered cancer therapeutics to the tumor microenvironment. Because αvβ3 and α5β1 are not expressed on normal (i.e., nonactivated) cells, targeting these integrins in man should provide an opportunity to selectively impair the function of multiple activated cell types within the tumor microenvironment, allowing for dose escalation with limited off-target effects and toxicity.

Although we found that the BsAb did not functionally outperform the combination of monospecific mAbs *in vitro* (Supplementary Fig. S6), a BsAb provides a more straightforward path for clinical development because it is a single therapeutic agent for which biodistribution, efficacy, and dose-limiting toxicity can be more cleanly determined compared with a combination of two individual mAbs that may differ in their pharmacokinetic, pharmacodynamic, and safety properties. In terms of quality of life, an additional potential benefit of a BsAb is the ability to inject patients with a single agent instead of two separate agents. Also, two antibodies delivered together may also produce steric hindrance effects, and it becomes complicated to determine an optimal safe and effective dose for each antibody. From a biological perspective, a BsAb may provide a functional benefit if both antigens are present on a single cell. To evaluate the biological importance of targeting α5β1 ± αvβ3 *in vivo*, we used the approach to knock down FN itself, as well as integrins α5 and β3, in CAFs before injection into mice. This strategy allowed us to selectively perturb FN and integrin function on the CAFs without affecting tumor cells that express these proteins and avoided complications associated with delivering multiple agents *in vivo* that may have significantly different pharmacokinetic profiles.

Our study approaches CAF–tumor cross-talk from a less mainstream angle that focuses on how CAFs might enable stem-like abilities of tumor cells. In contrast to the long-term impact of CAFs on tumor progression, our study relates to the ability of CAFs to provide individual tumor cells with supportive factors and ECM that allow them to become more stem-like to overcome “isolation stresses” (e.g., loss of cell–cell and cell–matrix contacts, nutrient or oxygen scarcity, or immune surveillance). This relationship may enhance the ability of a single tumor cell to gain cancer stem cell–like stress tolerance and invasive behavior that facilitates the establishment of new colonies within the pancreas, in the circulation, or at metastatic outposts. This point of view is unique compared with studies that explore the contributions of CAFs during later stages of cancer when cancer cells are already surrounded by a well-developed tumor microenvironment.

Although approaches to impair tumor initiation may not be appreciated when the growth of a primary tumor is the primary readout, our *in vitro* and *in vivo* models include challenges that mimic the critical steps involved in tumor initiation. Our findings agree with previous studies identifying FN as a key component of CAF function, in which knockdown of FN, β3, or α5 produced a significant effect on tumor cell invasion through ECM proteins *in vitro* (44). Indeed, CAFs produce ECM and soluble factors that strongly influence a tumor microenvironment, and integrins are ECM receptors utilized by all cell types to interact with and respond to specific forms of ECM (4, 45). In contrast to these studies, our new data show how CAFs can contribute to a somewhat different step of tumor progression, i.e., the ability of individual cancer cells to overcome isolation stress during tumor initiation or establishment of a growing tumor colony within an otherwise normal tissue environment that does not contain aberrant ECM. We propose that CAF enhancement of tumor initiation is relevant for scenarios that lack available therapeutic interventions. For example, circulating tumor cells in the bloodstream or disseminated tumor cells during metastasis must find a way to survive within environments that are inhospitable and prone to recognition by immune cells. As such, we propose that the contribution of CAFs to promote tumor initiation will involve a unique set of soluble factors and secreted matrix proteins that might not overlap with the role of CAFs that has been reported to support cancer cell growth in 2D cultures or invasion through ECM matrices *in vitro*.

Although the focus of our work is currently limited to pancreatic cancer, a disease for which few targeted therapeutics have produced clinical benefit, our findings might extend to additional cancer types because an individual circulating or disseminated tumor cell may utilize tissue-resident fibroblasts in the liver, lung, or lymph nodes during colonization of these sites during metastatic spread of pancreatic cancer. Similarly, other pathologic conditions that involve fibrosis might respond well to the blockade of FN–integrin interactions that provide a scaffold for the escalation of a chronic fibrotic response. Indeed, we show that targeting integrins αvβ3/α5β1 with the BsAb can suppress the generation of fibrotic ECM by primary fibroblasts isolated from patients with chronic lung or liver fibrosis.

## Supplementary Material

Supplementary Figure S1 Validation of KP4-luc orthotopic pancreatic cancer model

Supplementary Figure S2 FN and COL co-patterning requires FN

Supplementary Figure S3 Effect of αvβ3/α5β1 bispecific antibody on ECM assembly

Supplementary Figure S4 Effect of BsAb on primary fibroblasts isolated from patients with fibrotic disease

Supplementary Figure S5 BsAb treatment does not impact CAF mRNA expression of ECM proteins

Supplementary Figure S6 Comparison of bispecific antibody vs. combination of monoclonal antibodies

Supplementary Table S1 siRNAs used in this study

Supplementary Table S2 Integrin antibodies used in this study

Supplementary Table S3 Custom primer sequences for qPCR
