# Supplementary material for: Targeting Pancreatic Cancer Cell Stemness by Blocking Fibronectin-Binding Integrins on Cancer-Associated Fibroblasts
Source: Cancer Res Commun. 2025 Jan 31;5(1):195–208. doi: 10.1158/2767-9764.CRC-24-0491 (PMC11783622; doi:10.1158/2767-9764.CRC-24-0491)
Supplement: Supplementary Figure S1 — Validation of KP4-luc orthotopic pancreatic cancer model [file crc-24-0491_supplementary_figure_s1_suppsf1.pptx]

## Slide 1
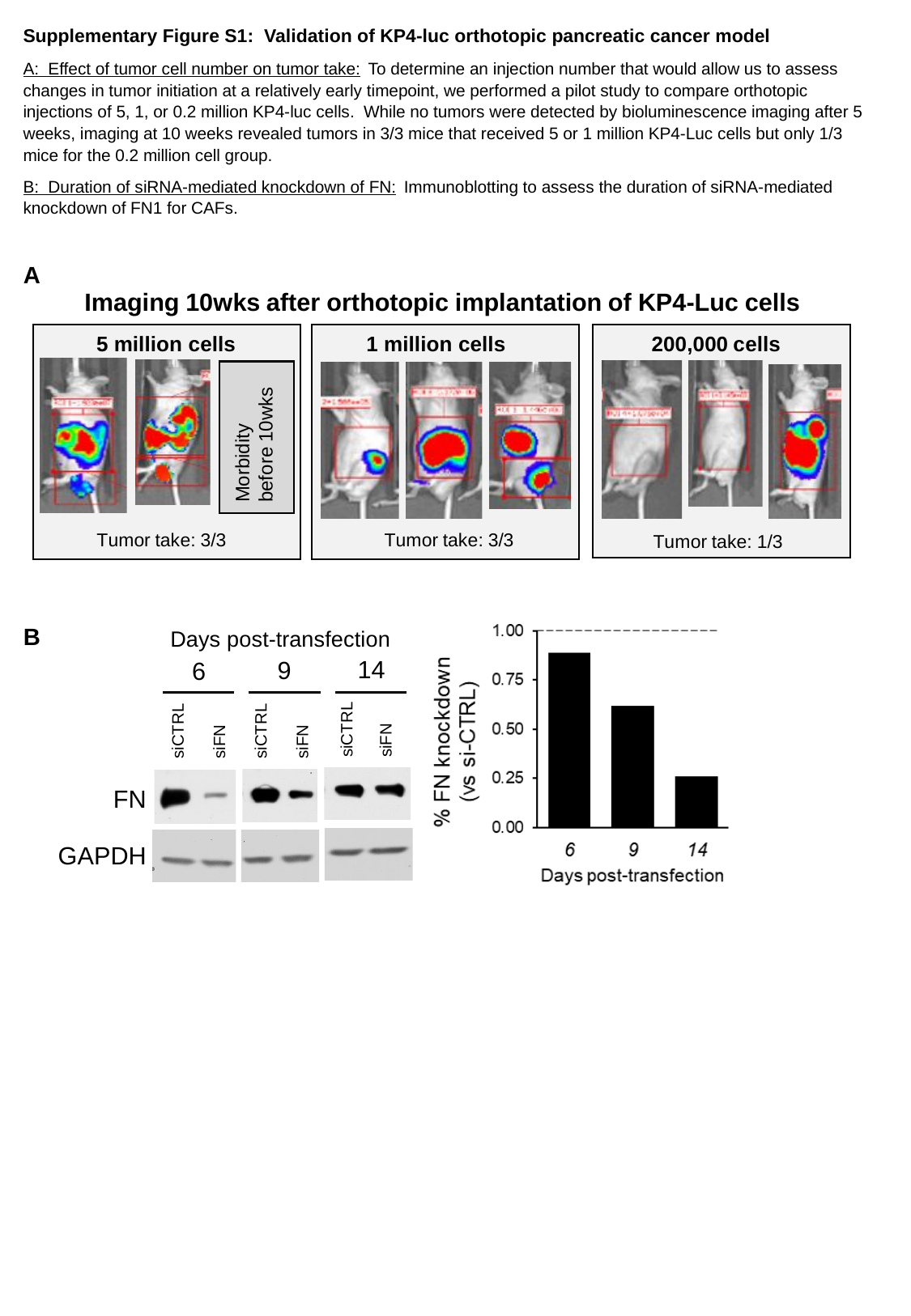

Supplementary Figure S1: Validation of KP4-luc orthotopic pancreatic cancer model
A: Effect of tumor cell number on tumor take: To determine an injection number that would allow us to assess changes in tumor initiation at a relatively early timepoint, we performed a pilot study to compare orthotopic injections of 5, 1, or 0.2 million KP4-luc cells. While no tumors were detected by bioluminescence imaging after 5 weeks, imaging at 10 weeks revealed tumors in 3/3 mice that received 5 or 1 million KP4-Luc cells but only 1/3 mice for the 0.2 million cell group.
B: Duration of siRNA-mediated knockdown of FN: Immunoblotting to assess the duration of siRNA-mediated knockdown of FN1 for CAFs.
A
B
