# Supplementary material for: Targeting Pancreatic Cancer Cell Stemness by Blocking Fibronectin-Binding Integrins on Cancer-Associated Fibroblasts
Source: Cancer Res Commun. 2025 Jan 31;5(1):195–208. doi: 10.1158/2767-9764.CRC-24-0491 (PMC11783622; doi:10.1158/2767-9764.CRC-24-0491)
Supplement: Supplementary Figure S2 — FN and COL co-patterning requires FN [file crc-24-0491_supplementary_figure_s2_suppsf2.pptx]

## Slide 1
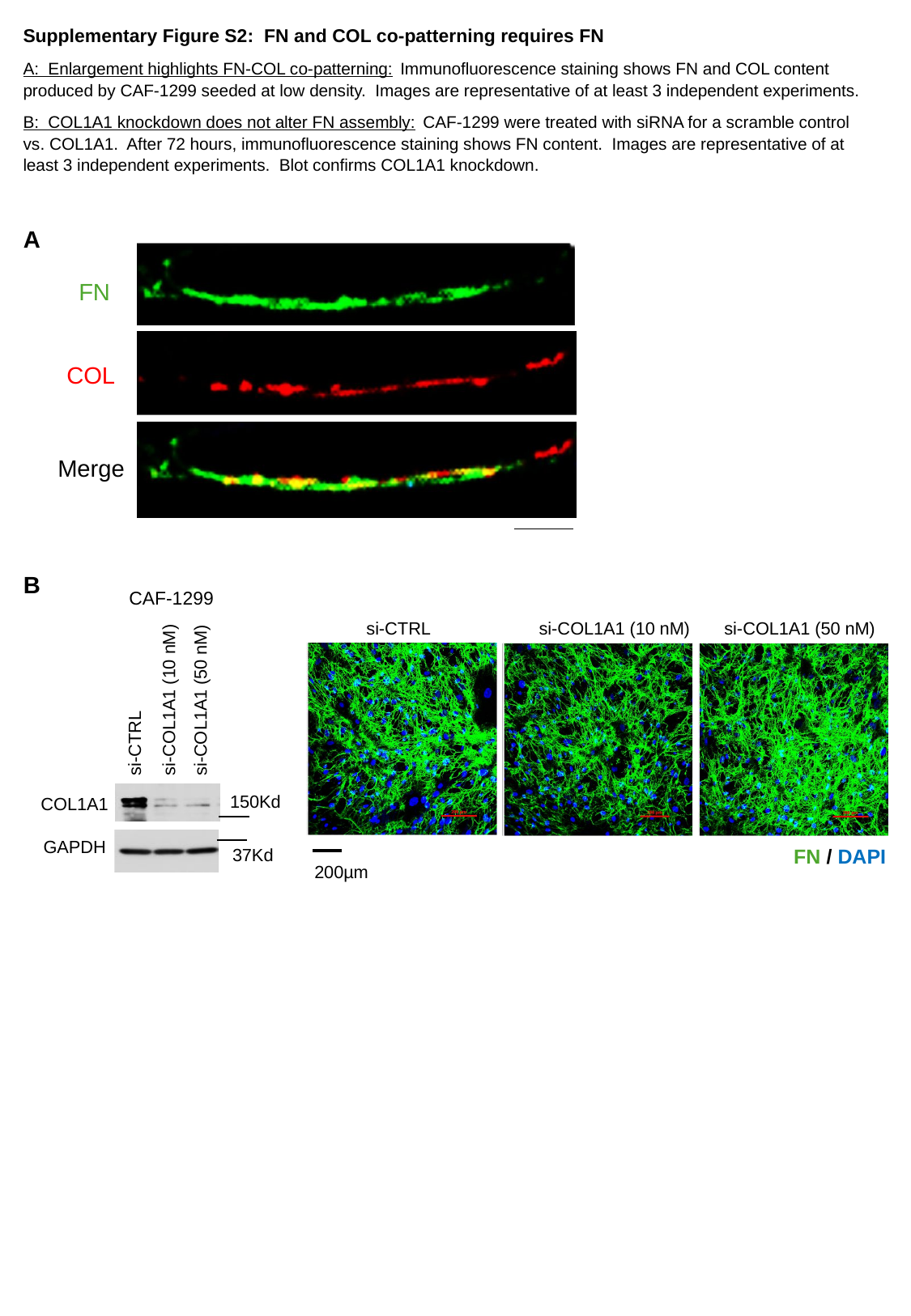

Supplementary Figure S2: FN and COL co-patterning requires FN
A: Enlargement highlights FN-COL co-patterning: Immunofluorescence staining shows FN and COL content produced by CAF-1299 seeded at low density. Images are representative of at least 3 independent experiments.
B: COL1A1 knockdown does not alter FN assembly: CAF-1299 were treated with siRNA for a scramble control vs. COL1A1. After 72 hours, immunofluorescence staining shows FN content. Images are representative of at least 3 independent experiments. Blot confirms COL1A1 knockdown.
A
Merge
COL
FN
B
CAF-1299
si-COL1A1 (10 nM)
si-COL1A1 (50 nM)
si-CTRL
COL1A1
GAPDH
150Kd
37Kd
si-COL1A1 (10 nM)
si-COL1A1 (50 nM)
si-CTRL
FN / DAPI
200µm
