# Supplementary material for: Targeting Pancreatic Cancer Cell Stemness by Blocking Fibronectin-Binding Integrins on Cancer-Associated Fibroblasts
Source: Cancer Res Commun. 2025 Jan 31;5(1):195–208. doi: 10.1158/2767-9764.CRC-24-0491 (PMC11783622; doi:10.1158/2767-9764.CRC-24-0491)
Supplement: Supplementary Figure S3 — Effect of αvβ3/α5β1 bispecific antibody on ECM assembly [file crc-24-0491_supplementary_figure_s3_suppsf3.pptx]

## Slide 1
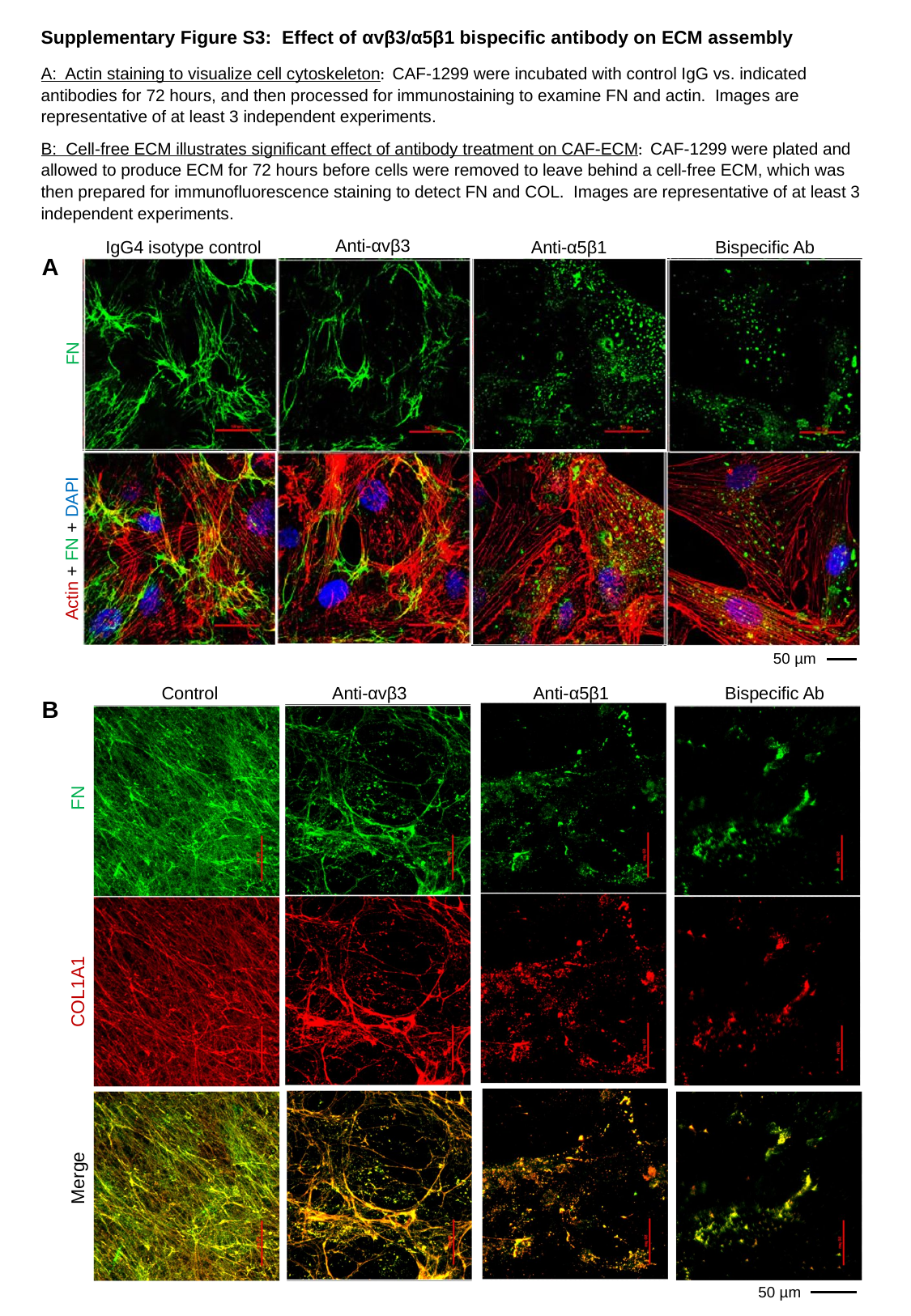

Supplementary Figure S3: Effect of αvβ3/α5β1 bispecific antibody on ECM assembly
A: Actin staining to visualize cell cytoskeleton: CAF-1299 were incubated with control IgG vs. indicated antibodies for 72 hours, and then processed for immunostaining to examine FN and actin. Images are representative of at least 3 independent experiments.
B: Cell-free ECM illustrates significant effect of antibody treatment on CAF-ECM: CAF-1299 were plated and allowed to produce ECM for 72 hours before cells were removed to leave behind a cell-free ECM, which was then prepared for immunofluorescence staining to detect FN and COL. Images are representative of at least 3 independent experiments.
Anti-αvβ3
IgG4 isotype control
Anti-α5β1
Bispecific Ab
A
FN
Actin + FN + DAPI
50 µm
Anti-α5β1
Anti-αvβ3
Bispecific Ab
Control
FN
COL1A1
Merge
50 µm
B
