# Supplementary material for: Targeting Pancreatic Cancer Cell Stemness by Blocking Fibronectin-Binding Integrins on Cancer-Associated Fibroblasts
Source: Cancer Res Commun. 2025 Jan 31;5(1):195–208. doi: 10.1158/2767-9764.CRC-24-0491 (PMC11783622; doi:10.1158/2767-9764.CRC-24-0491)
Supplement: Supplementary Figure S4 — Effect of BsAb on primary fibroblasts isolated from patients with fibrotic disease [file crc-24-0491_supplementary_figure_s4_suppsf4.pptx]

## Slide 1
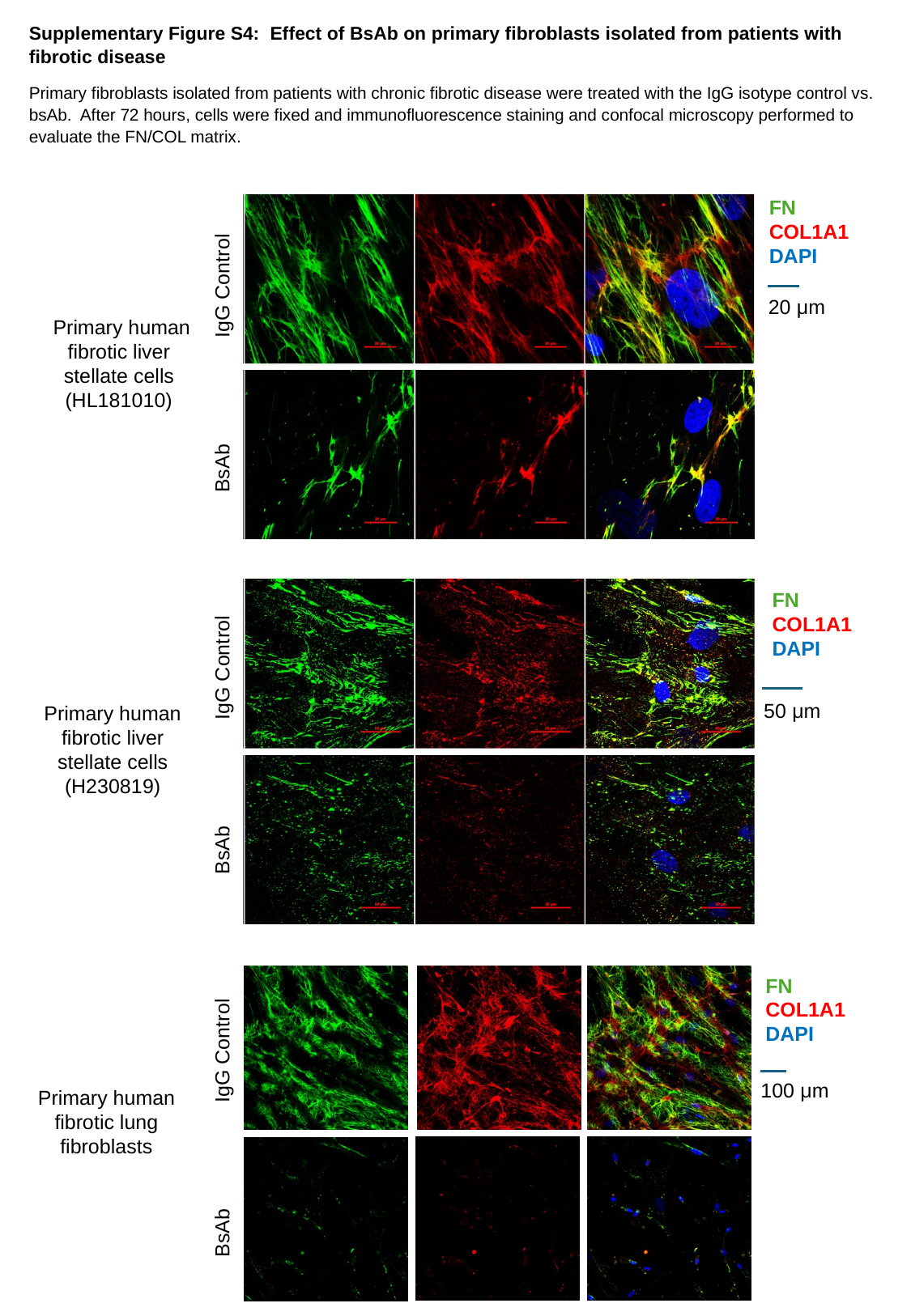

Supplementary Figure S4: Effect of BsAb on primary fibroblasts isolated from patients with fibrotic disease
Primary fibroblasts isolated from patients with chronic fibrotic disease were treated with the IgG isotype control vs. bsAb. After 72 hours, cells were fixed and immunofluorescence staining and confocal microscopy performed to evaluate the FN/COL matrix.
FN
COL1A1
DAPI
IgG Control
20 μm
 Primary human fibrotic liver stellate cells (HL181010)
BsAb
FN
COL1A1
DAPI
IgG Control
50 μm
Primary human fibrotic liver stellate cells (H230819)
BsAb
FN
COL1A1
DAPI
IgG Control
100 μm
Primary human fibrotic lung fibroblasts
BsAb
