# Supplementary material for: Targeting Pancreatic Cancer Cell Stemness by Blocking Fibronectin-Binding Integrins on Cancer-Associated Fibroblasts
Source: Cancer Res Commun. 2025 Jan 31;5(1):195–208. doi: 10.1158/2767-9764.CRC-24-0491 (PMC11783622; doi:10.1158/2767-9764.CRC-24-0491)
Supplement: Supplementary Figure S5 — BsAb treatment does not impact CAF mRNA expression of ECM proteins [file crc-24-0491_supplementary_figure_s5_suppsf5.pptx]

## Slide 1
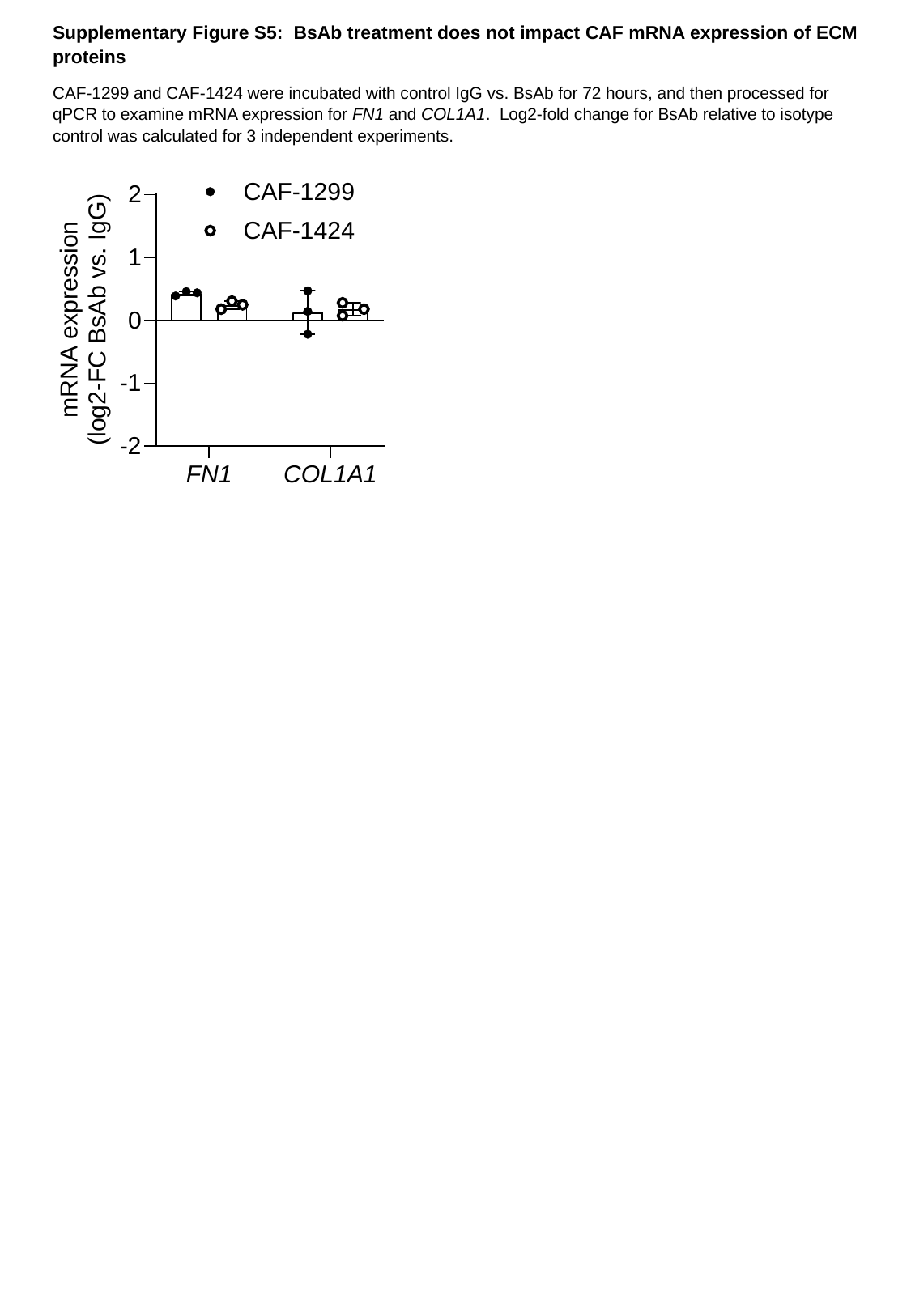

Supplementary Figure S5: BsAb treatment does not impact CAF mRNA expression of ECM proteins
CAF-1299 and CAF-1424 were incubated with control IgG vs. BsAb for 72 hours, and then processed for qPCR to examine mRNA expression for FN1 and COL1A1. Log2-fold change for BsAb relative to isotype control was calculated for 3 independent experiments.
