# Supplementary material for: Targeting Pancreatic Cancer Cell Stemness by Blocking Fibronectin-Binding Integrins on Cancer-Associated Fibroblasts
Source: Cancer Res Commun. 2025 Jan 31;5(1):195–208. doi: 10.1158/2767-9764.CRC-24-0491 (PMC11783622; doi:10.1158/2767-9764.CRC-24-0491)
Supplement: Supplementary Figure S6 — Comparison of bispecific antibody vs. combination of monoclonal antibodies [file crc-24-0491_supplementary_figure_s6_suppsf6.pptx]

## Slide 1
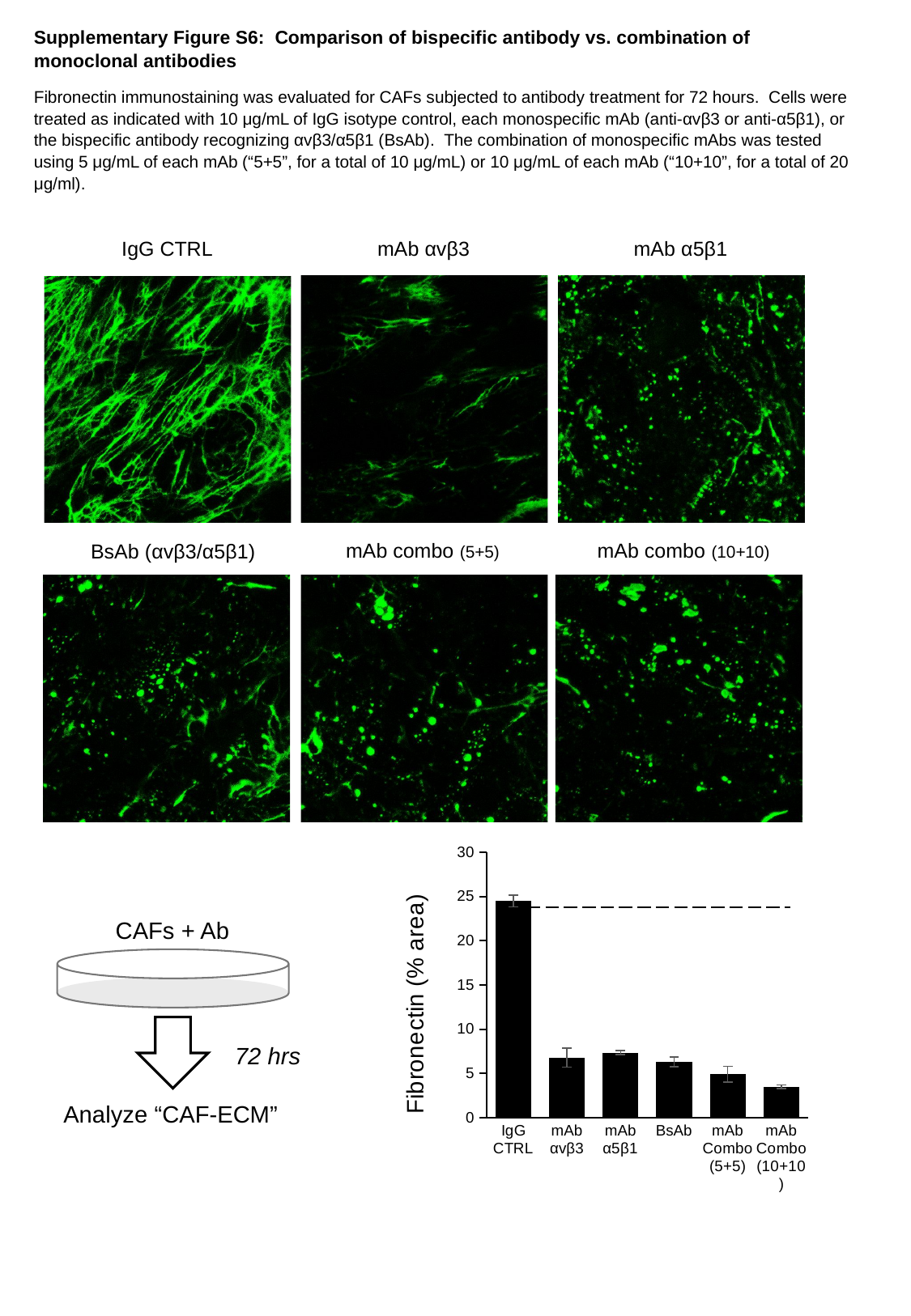

Supplementary Figure S6: Comparison of bispecific antibody vs. combination of monoclonal antibodies
Fibronectin immunostaining was evaluated for CAFs subjected to antibody treatment for 72 hours. Cells were treated as indicated with 10 μg/mL of IgG isotype control, each monospecific mAb (anti-αvβ3 or anti-α5β1), or the bispecific antibody recognizing αvβ3/α5β1 (BsAb). The combination of monospecific mAbs was tested using 5 μg/mL of each mAb (“5+5”, for a total of 10 μg/mL) or 10 μg/mL of each mAb (“10+10”, for a total of 20 μg/ml).
IgG CTRL
mAb αvβ3
mAb α5β1
mAb combo (10+10)
mAb combo (5+5)
BsAb (αvβ3/α5β1)
Green = FN
### Chart
| Category | Green |
|---|---|
| IgG CTRL | 24.4895 |
| mAb αvβ3 | 6.774 |
| mAb α5β1 | 7.333666666666666 |
| BsAb | 6.301 |
| mAb Combo (5+5) | 4.8975 |
| mAb Combo (10+10) | 3.49 |CAFs + Ab
72 hrs
Analyze “CAF-ECM”
